# Cutaneous T-cell lymphomas with pathogenic somatic mutations and absence of detectable clonal T-cell receptor gene rearrangement: two case reports

**DOI:** 10.1186/s13000-020-01022-x

**Published:** 2020-09-28

**Authors:** Rebecca Rojansky, Sebastian Fernandez-Pol, Erica Wang, Kerri E. Rieger, Roberto A. Novoa, James L. Zehnder, Christian A. Kunder, Youn H. Kim, Michael S. Khodadoust, Ryanne A. Brown

**Affiliations:** 1 Department of Pathology, Stanford Medicine, Stanford, CA 94305 USA; 2 Department of Dermatology, Stanford Medicine, Stanford, CA 94305 USA; 3 Division of Hematology, Department of Medicine, Stanford Medicine, Stanford, CA 94305 USA; 4 Division of Oncology, Department of Medicine, Stanford Medicine, Stanford, CA 94305 USA; 5 Department of Pathology, Veterans Affairs Palo Alto Health Care System, 3375 Hillview Ave, Room 1821, Palo Alto, CA 94304-1204 USA

**Keywords:** Lymphoma, Mycosis fungoides, T-cell receptor, Clonality, Next-generation sequencing, case report

## Abstract

**Background:**

Cutaneous T-cell lymphomas (CTCL) are a heterogeneous group of extranodal non-Hodgkin lymphomas for which diagnosis can be challenging given the potential for overlap with inflammatory dermatoses. Current diagnostic criteria for CTCL incorporate clinical and histopathologic findings as well as results of T-cell receptor (TCR) gene sequencing. Molecular interrogation of TCR genes, *TRG* and *TRB*, has proven to be a critical tool for confirming diagnoses of CTCL and for disease tracking after initiation of therapy or after stem cell transplant. Methods for confirming a diagnosis of lymphoma in the absence of TCR gene clonality are lacking. We present two patients with CTCL with pathogenic somatic mutations in the absence of *TRG* and *TRB* clonality.

**Case presentations:**

Case 1: A 38-year-old male had a 19-year history of a diffuse skin rash with papulosquamous, granulomatous, and verrucous features and progressive ulcerated plaques and tumors demonstrating an atypical CD4+ T-cell infiltrate with expression of cytotoxic markers CD56, TIA-1, granzyme, and perforin on histopathology. No definitive evidence for T-cell clonality was detected by conventional PCR of 6 biopsies or by next-generation sequencing (NGS) of 14 biopsies. Somatic mutational profiling of a skin biopsy revealed pathogenic mutations in *PIKC3D* and *TERT* promoter hotspots, confirming the presence of a clonal process. Case 2: A 69-year-old male with a 13-year history of progressive, diffuse hypertrophic and eroded plaques showed an atypical CD4+ T-cell infiltrate with subset expression of TIA-1 and granzyme on histopathology. No TCR clonality was detected by TCR-NGS of 6 biopsies. Somatic mutational profiling of a skin biopsy detected a pathogenic mutation in *TP53*, confirming the presence of a clonal process.

**Conclusions:**

These cases highlight how detection of pathogenic somatic mutations can confirm a diagnosis of lymphoma in a clinically and histopathologically suspicious cutaneous lymphoid proliferation without detectable TCR clonality.

## Background

Cutaneous T-cell lymphomas (CTCL) account for the majority (up to 75%) of primary cutaneous lymphomas and are designated according to the updated WHO-EORTC 2018 classification [1]. Diagnosis of CTCL requires integration of clinical, histopathologic, and molecular findings, the latter of which includes detection of clonal T-cell receptor (TCR) gene rearrangements via interrogation of TCRβ (*TRB*) and TCRγ (*TRG*) genes. A majority of mature T-cell malignancies express membranous CD3 in complex with TCR [2]. TCR gene rearrangement occurs exclusively during thymic T-cell development to facilitate diverse antigen recognition and not in mature circulating T-cells, so the detection of TCR monoclonality generally supports a clonal process such as T-cell lymphoma over an inflammatory disorder [3, 4]. TCR clonality can also indicate T-cell origin in a lymphoid process of unknown lineage, reveal a clonal T-cell proliferation in the background of another malignant process, serve as a marker for minimal residual disease (MRD) testing, or indicate common origin of distinct lesions [5]. Even when present, however, the definition and significance of TCR clonality remains subject to interpretation, especially when referring to the results of conventional polymerase chain reaction (PCR)-based clonality testing [6]. Some inflammatory dermatoses with benign clinical course such as pityriasis lichenoides have been shown to demonstrate TCR clonality [7]. Therefore, while TCR clonality is a crucial part of diagnosing peripheral T-cell lymphomas, it must be interpreted carefully and in the context of the entire clinical and histopathologic picture. Here we present two CTCL cases from our institution with polyclonal TCR by conventional PCR and high throughput next-generation sequencing (NGS). Both cases demonstrated clonal pathogenic somatic mutations, confirming their clonal origin and thus the diagnosis of lymphoma.

## Case presentations

### Case 1

**Fig. 1:**
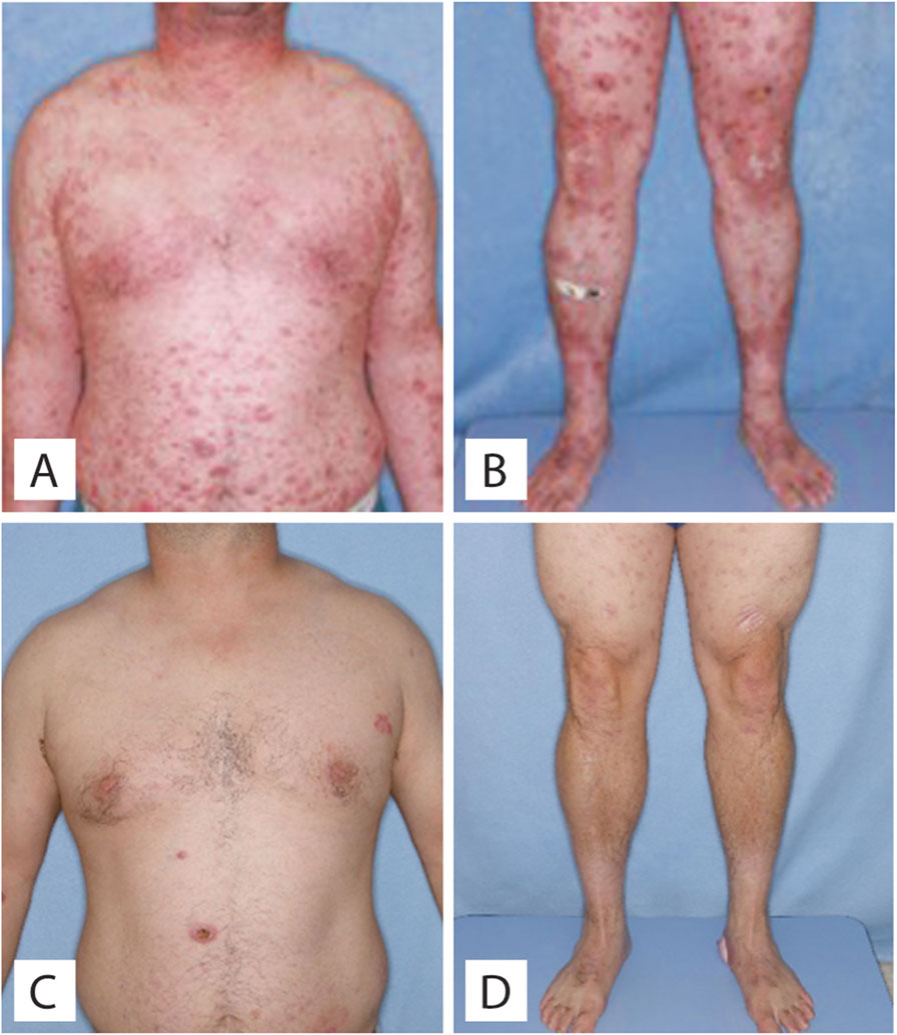
Clinical photos of Case 1. **a**-**b**, Upon presentation at our institution. Erythematous scaly flat-topped papules and plaques. **c**-**d** Improvement after therapy

**Fig. 2:**
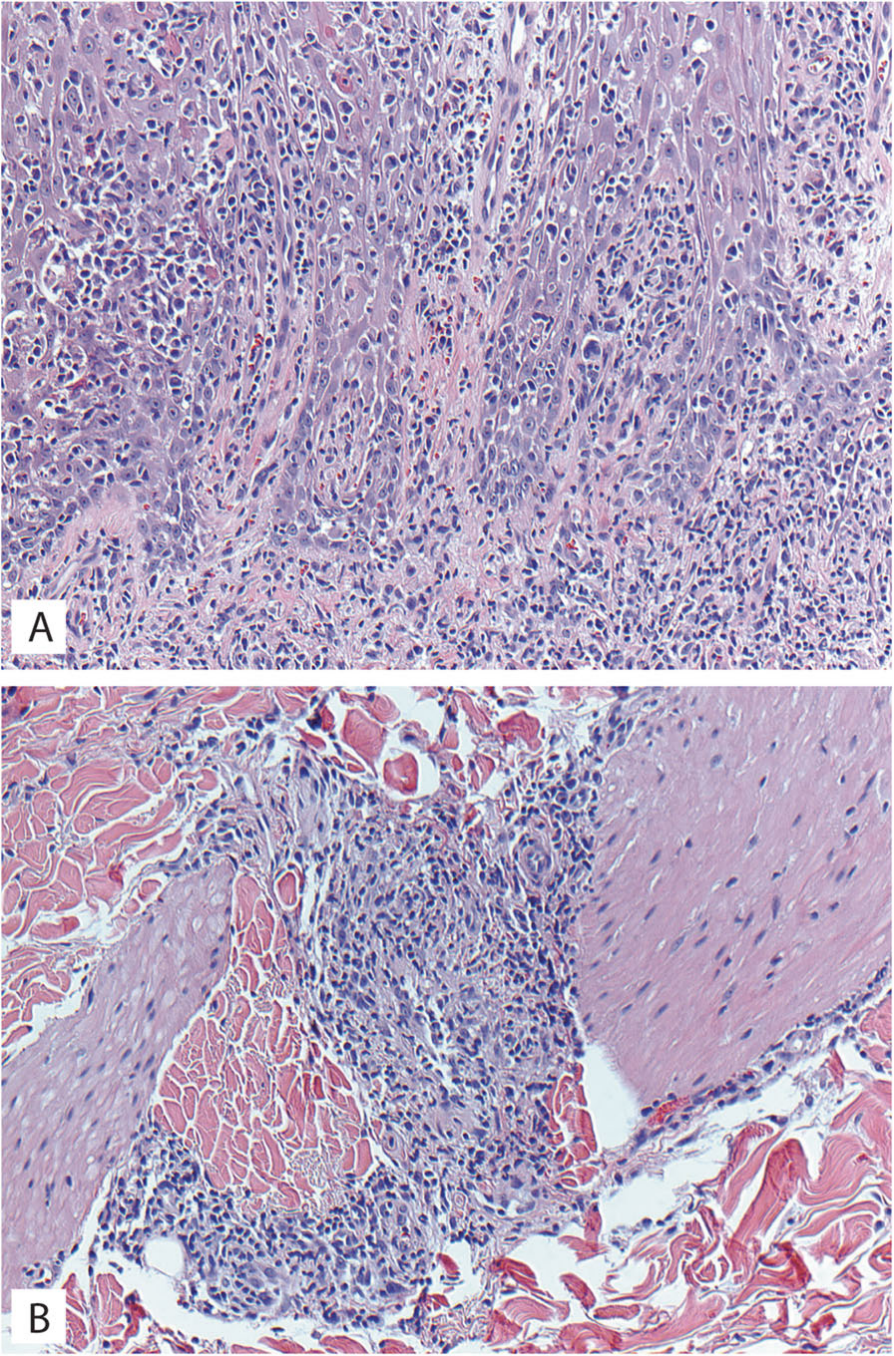
Microscopic images of Case 1 histopathology. **a** Biopsy of left thigh lesion showing dense lichenoid and epidermotropic infiltrate of small to medium size lymphocytes with irregular nuclear contours; H&E 200X. **b** Biopsy of left thigh lesion showing a nodular granulomatous and lymphocytic dermal infiltrate centered on a vessel; H&E 200X

**Fig. 3:**
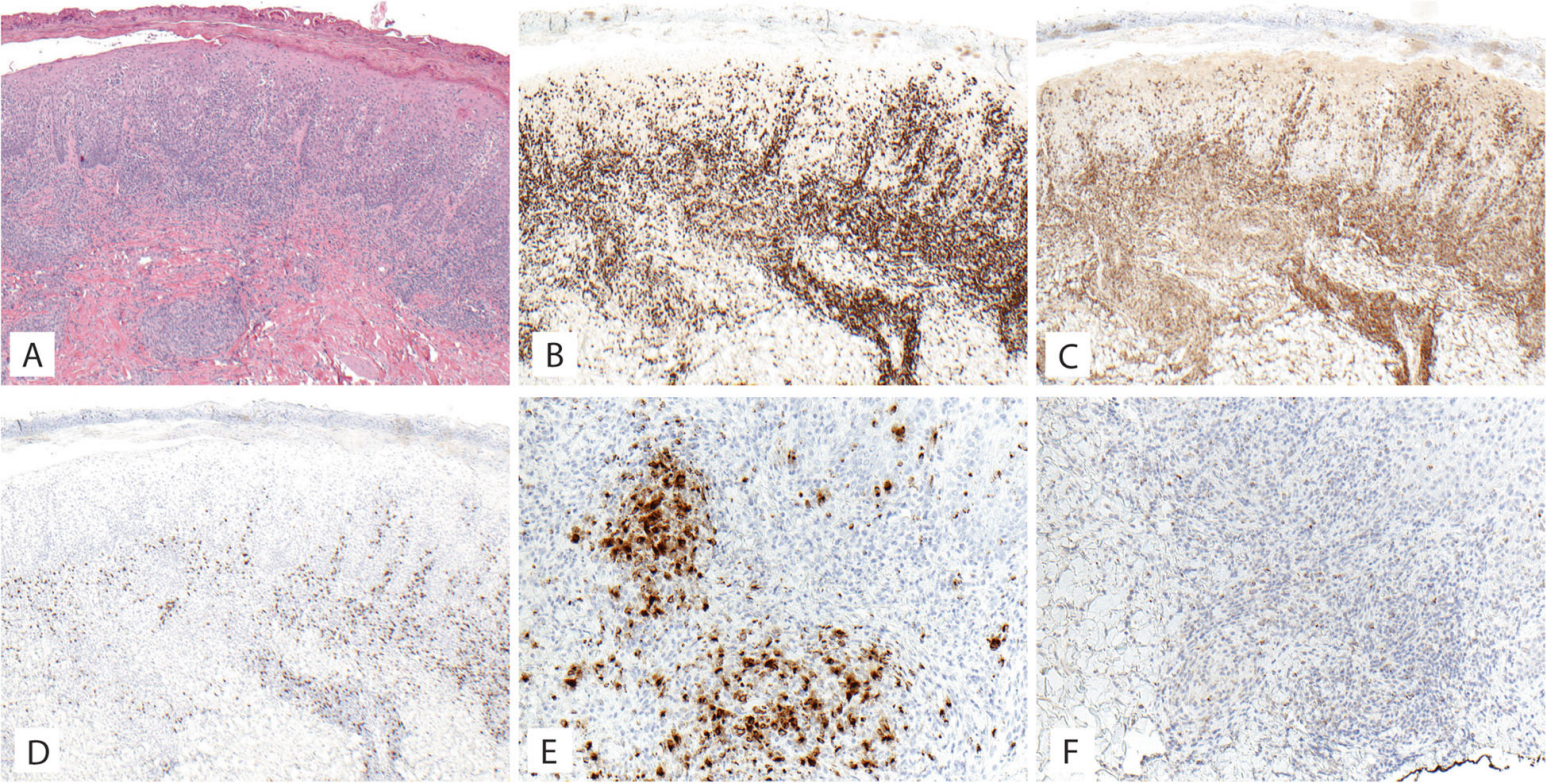
Microscopic images of Case 1 biopsy of left thigh lesion. **a**, Lichenoid and epidermal infiltrate; H&E 40X. **b** CD3 immunostain; 40X. **c** CD4 immunostain; 40X. **d** CD8 immunostain; 40X. **e** granzyme immunostain; 40X. **f** TIA1 immunostain; 40X

**Fig. 4:**
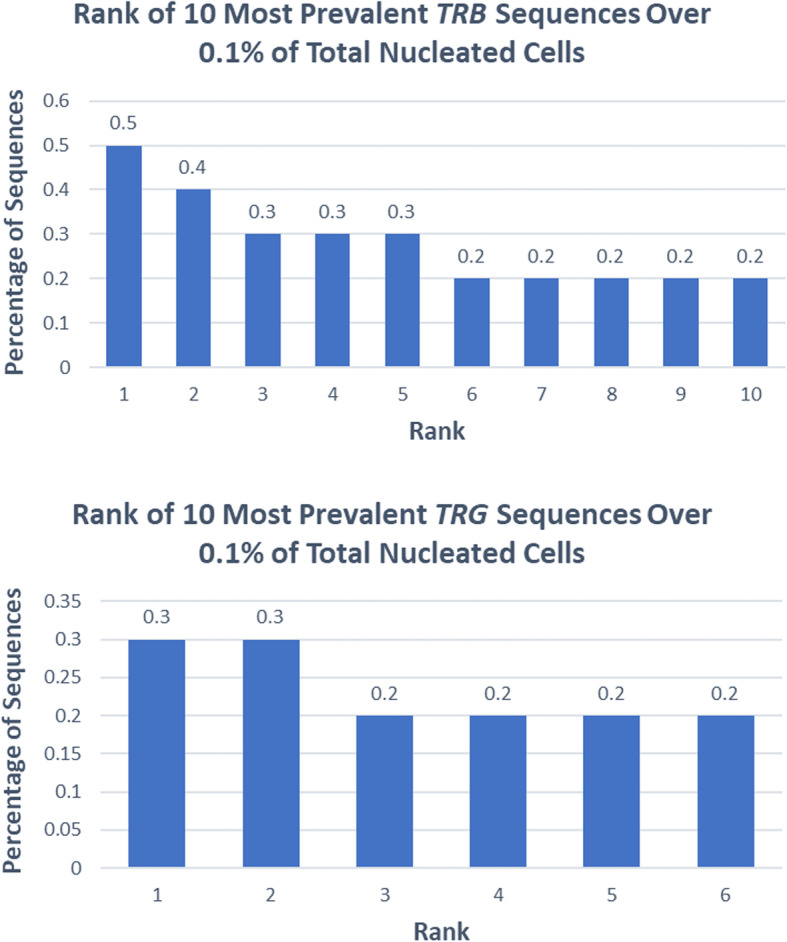
Case 1. Rank of most prevalent sequences over 0.1% by next-generation sequencing of *TRB* (top) and *TRG* (bottom) for one skin biopsy

**Fig. 5:**
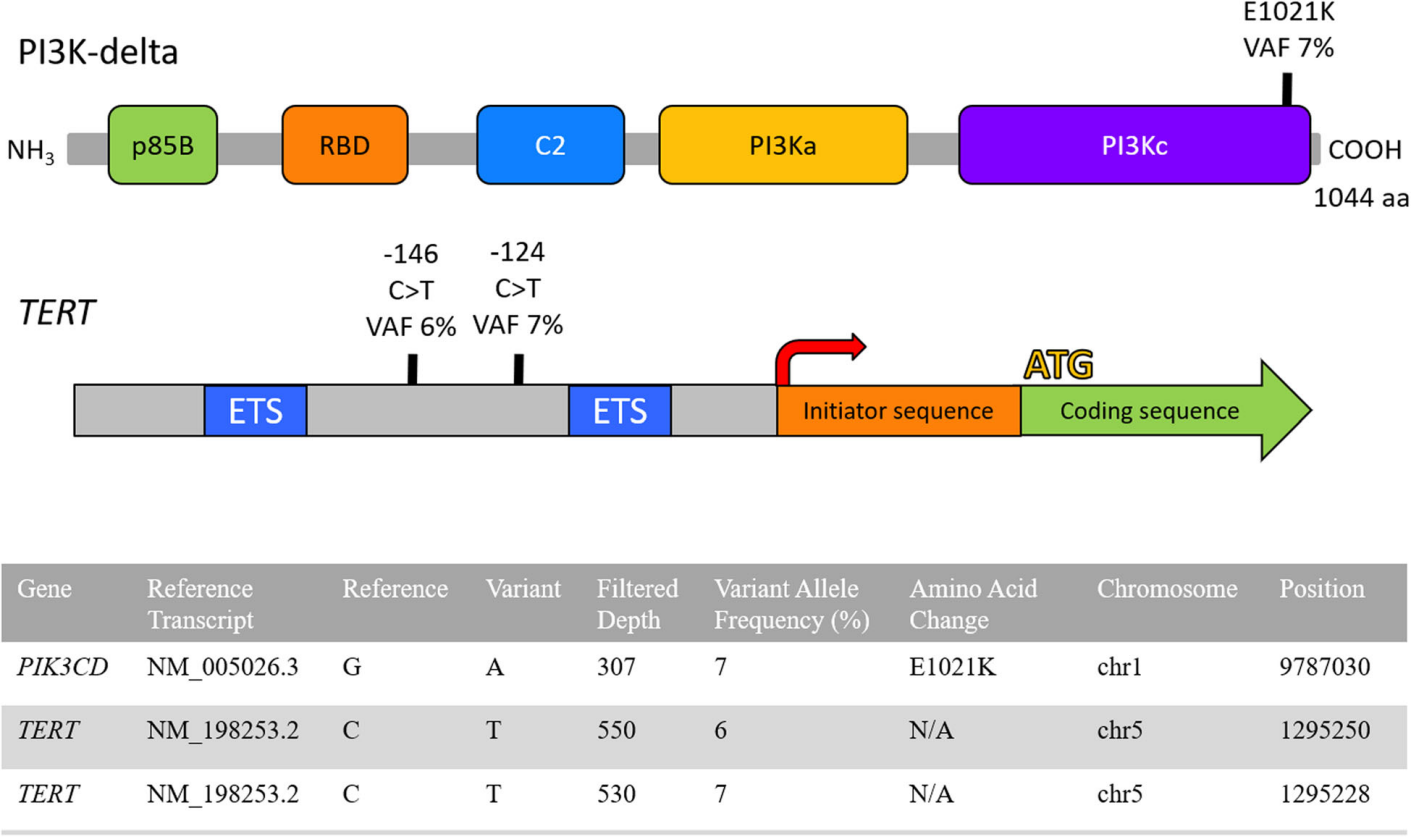
Candidate mutations in patient 1. Top: Structure of PI3K-delta protein with functional domains and point mutation at position 1021 located in the catalytic domain. p85B = p85 binding domain, RBD = ras binding domain, C2 = C2 domain present in class I beta and delta PI3Ks, PI3Ka = accessory domain, PI3Kc = catalytic domain. Middle: Structure of *TERT* gene and upstream regulatory region showing two point mutations identified in the *TERT* promoter. Red arrow = transcription start site, ATG = coding sequence start site, ETS = ETS family transcription factor binding sites. Bottom: Candidate pathogenic mutations identified via targeted sequencing analysis of case 1 biopsy specimen by Heme-STAMP*.* Filtered depth = read depth of the variant allele

A 38-year-old previously healthy male presented to an outside institution at age 19 with an erythematous macular abdominal rash, which progressed to diffuse cutaneous involvement over 12 years with eventual development of polymorphic clinical features, including papulosquamous, granulomatous, and verrucous skin lesions with progressive ulcerated plaques and tumors (Fig. 1a-b). He showed some improvement with total skin electron beam therapy and localized radiotherapy but ultimately progressed. His disease was persistent despite numerous chemotherapeutic and immunomodulatory agents including romidepsin, pralatrexate, pralatrexate with bexarotene, bexarotene alone, and brentuximab vedotin. His biopsies (a total of 22) showed two distinct phenotypes: 1) lichenoid-like infiltrates composed primarily of lymphocytes and histiocytes with saw-toothing of rete ridges, colloid body formation, and variably present epidermotropic medium-sized atypical lymphocytes (Fig. 2a); or 2) a dense nodular granulomatous and lymphocytic dermal infiltrate (Fig. 2b). Immunohistochemistry demonstrated that the lymphocytic component was composed of CD3-positive, TCRß-expressing T-cells with a CD4:CD8 ratio of over 10:1, retained expression of CD2, CD5, and CD7, and CD30 expression in a minor subset. A subset of T-cells showed a cytotoxic profile with expression of CD56, TIA-1, granzyme, and perforin (Fig. 3). PD-1 was negative. Ebstein Barr virus (EBV) in situ hybridization was negative and immunohistochemistry for CD20 stained background B-cells. No definitive evidence for T-cell clonality was detected by conventional PCR of 6 biopsies and by NGS (Adaptive Biotechnologies, 1551 Eastlake Ave. E, Ste. 200, Seattle, WA 98102) of 14 biopsies (Fig. 4). Conventional PCR involved analysis for T-cell clonality and comparison of the clones using the BIOMED-2 primer set for TCRγ chain (dual TCR-PCR) [8]. Six fluorescently labeled BIOMED-2 primers in two tubes allowed for the detection of nearly all Vγ-Jγ combinations (Invivoscribe, Carlsbad, CA). After amplification, the PCR products were analyzed on an automated capillary electrophoresis system (ABI 3100, Applied Biosystems, Foster City, CA) with GeneScan software (Applied Biosystems). NGS-TCR by Adaptive involved qPCR amplification of *TRB* and *TRG* CDR3 (complementarity determining region 3), using tagged V- and J-gene primers, followed by sequencing of the amplified CDR3 regions. The PCR amplification was multiplexed with many V-forward and J-reverse primers (specific to *TRB* and *TRG*). The amplification bias was controlled by adjusting primer concentrations (based on their annealing properties) and adding to the PCR mixture a complete synthetic V(D)J template repertoire (with every possible V-J pair) at very low concentration to allow internal PCR calibration. After sequencing and enumerating all the TCR CDR3 regions, the residual bias was removed computationally. For a sequence to be considered dominant by this methodology, it must be ≥3% of all like sequences (*TRB* or *TRG*), ≥0.2% of the total nucleated cells in the sample, discontinuously distributed (≤5 sequences in the next decade of sequences by frequency), and carried by ≥40 estimated genome equivalents in the analyzed sample. TCR polyclonality was cughput sequencing was performed on his most recent biopsy using an in-house developed, clinically-validated NGS panel [Solid Tumor Actionable Mutation Panel for Hematopoietic and Lymphoid Malignancies (Heme-STAMP)] that covers 164 genes, either in part or fully, with the genes selected on the basis of their known impact as actionable targets of existing and emerging anti-cancer therapies, their prognostic features, and/or their mutation recurrence frequency across patients with known cancer types. The assay has a minimum analytic detection limit of at least 5% for single nucleotide variants and short insertion/deletions. The sequencing was performed on an Illumina® NextSeq system. Bioinformatic analysis was performed using a pipeline developed and validated for clinical specimens. No paired normal tissue was available for comparison. Heme-STAMP identified three pathogenic mutations and their variant allele frequencies (VAF), including one in *PIK3CD* (c.3061G > A, p.E1021K, NM_005026.3, VAF 7%), which encodes the delta subunit of phosphatidylinositol 3-kinase (PI3K) (Fig. 5). The PI3K pathway is one of the most commonly mutated pathways in cancer [9]. The E1021K mutation identified in this patient is an activating mutation in the kinase domain of the protein and is known to be associated with B cell lymphomas [10]. When present in the germline, this mutation is associated with a combined variable immunodeficiency disease known as activated PI3Kd syndrome, which can present with granulomatous skin lesions [11, 12]. Other mutations in the *PIK3CD* gene have also been identified in hepatosplenic T-cell lymphomas [13]. Two additional mutations were in *TERT* (NM_198253.2) promoter hotspots (c.-146C > T, VAF 6%; c.-124C > T, VAF 7%, also referred to in the literature as C250T and C228T, respectively) that lead to enhanced telomerase expression. Activating *TERT* promoter mutations are known to be associated with skin cancers including melanoma [14]. These pathogenic mutations support the clinical and histopathologic impression of CTCL. While awaiting possible allogeneic stem cell transplant, the patient has been stable or improving on pralatrexate (Fig. 1c-d), an antifolate chemotherapy considered standard of care for the treatment of CTCL [15, 16].

### Case 2

**Fig. 6:**
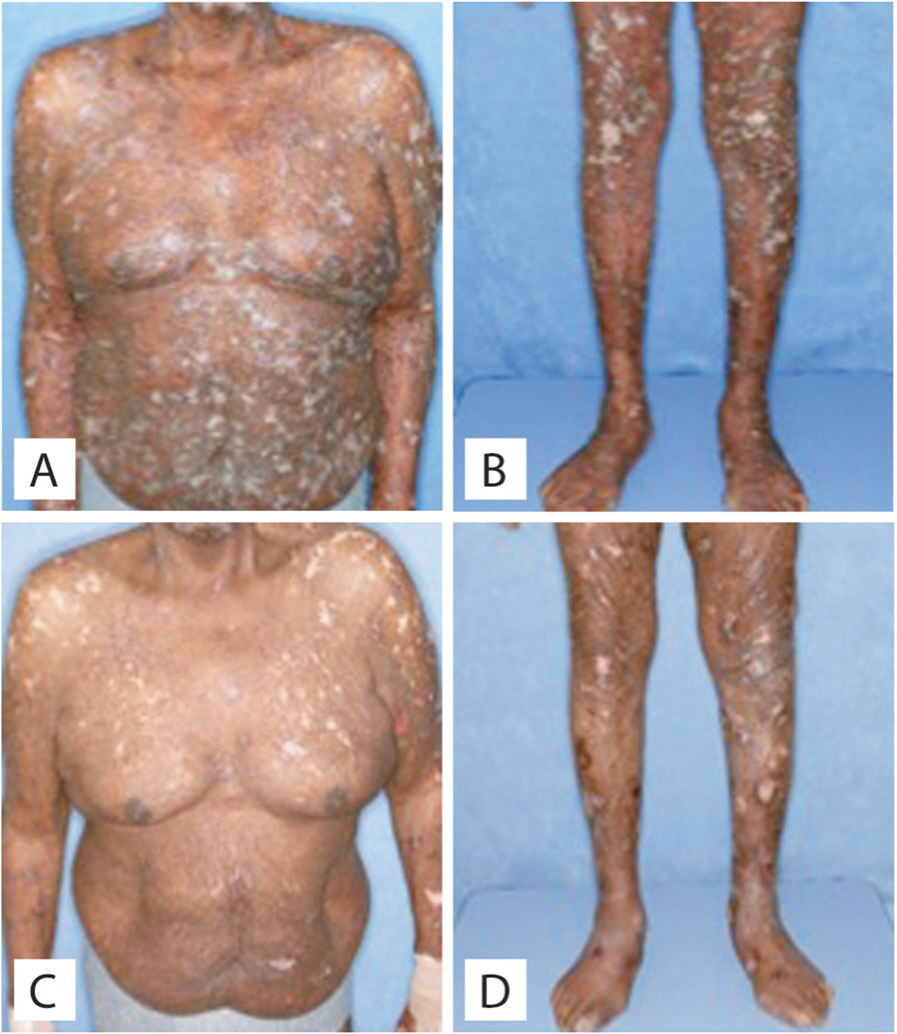
Clinical photos of Case 2. **a**-**b** Upon presentation at our institution. Diffuse erythematous hypertrophic and ulcerated papules and plaques. **c**-**d** Improvement after therapy

**Fig. 7:**
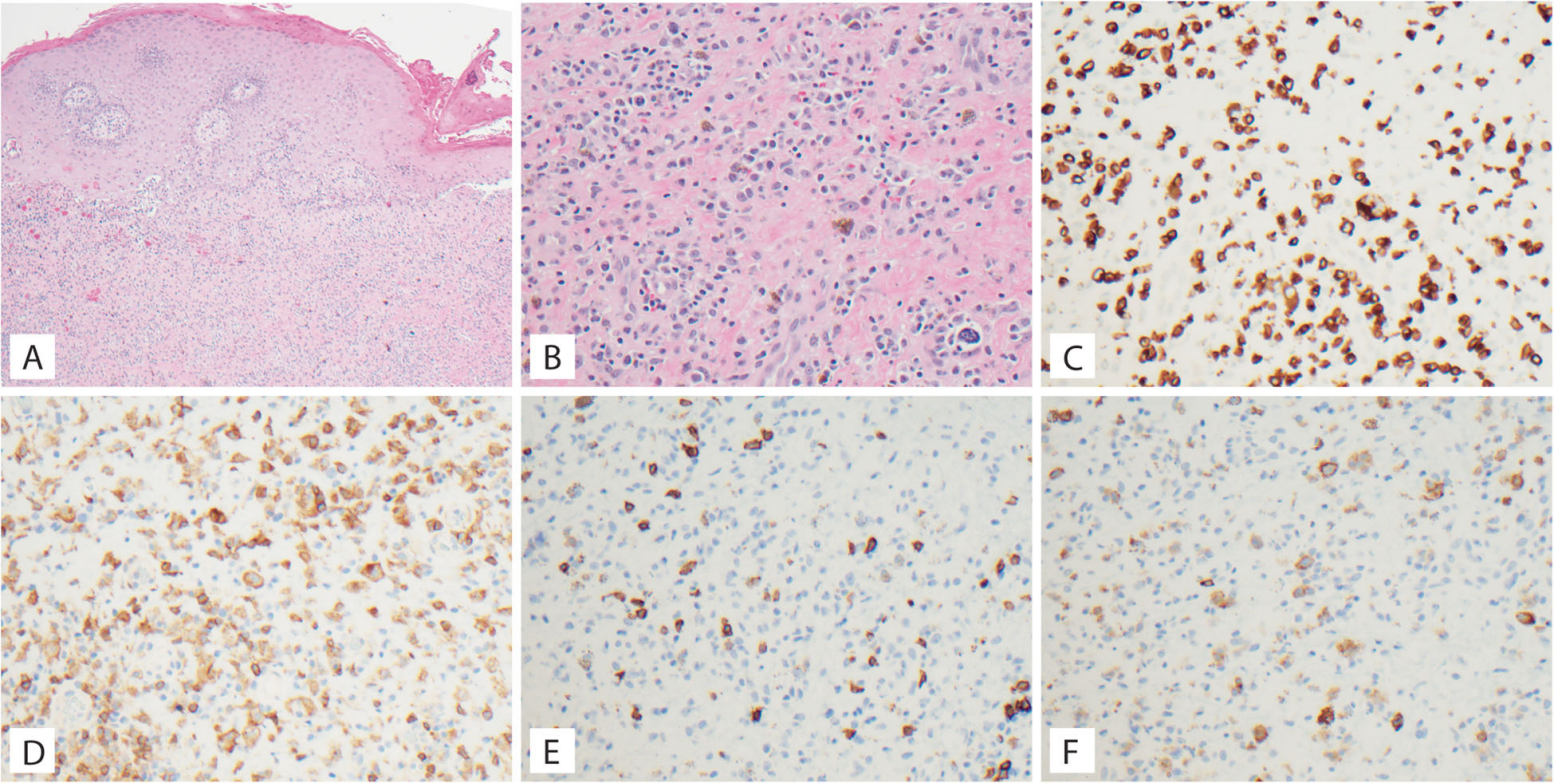
Microscopic images of Case 2 biopsy of left upper back biopsy. **a** Skin with a dermal and epidermotropic infiltrate of atypical lymphocytes with irregularly contoured nuclei; H&E 40X. **b** Higher power shows that a subset of cells is large in size; H&E 200X. **c** CD3 immunostain; 200X. **d** CD4 immunostain; 200X. **e** CD8 immunostain; 200X. **f** CD30 immunostain; 200X

**Fig. 8:**
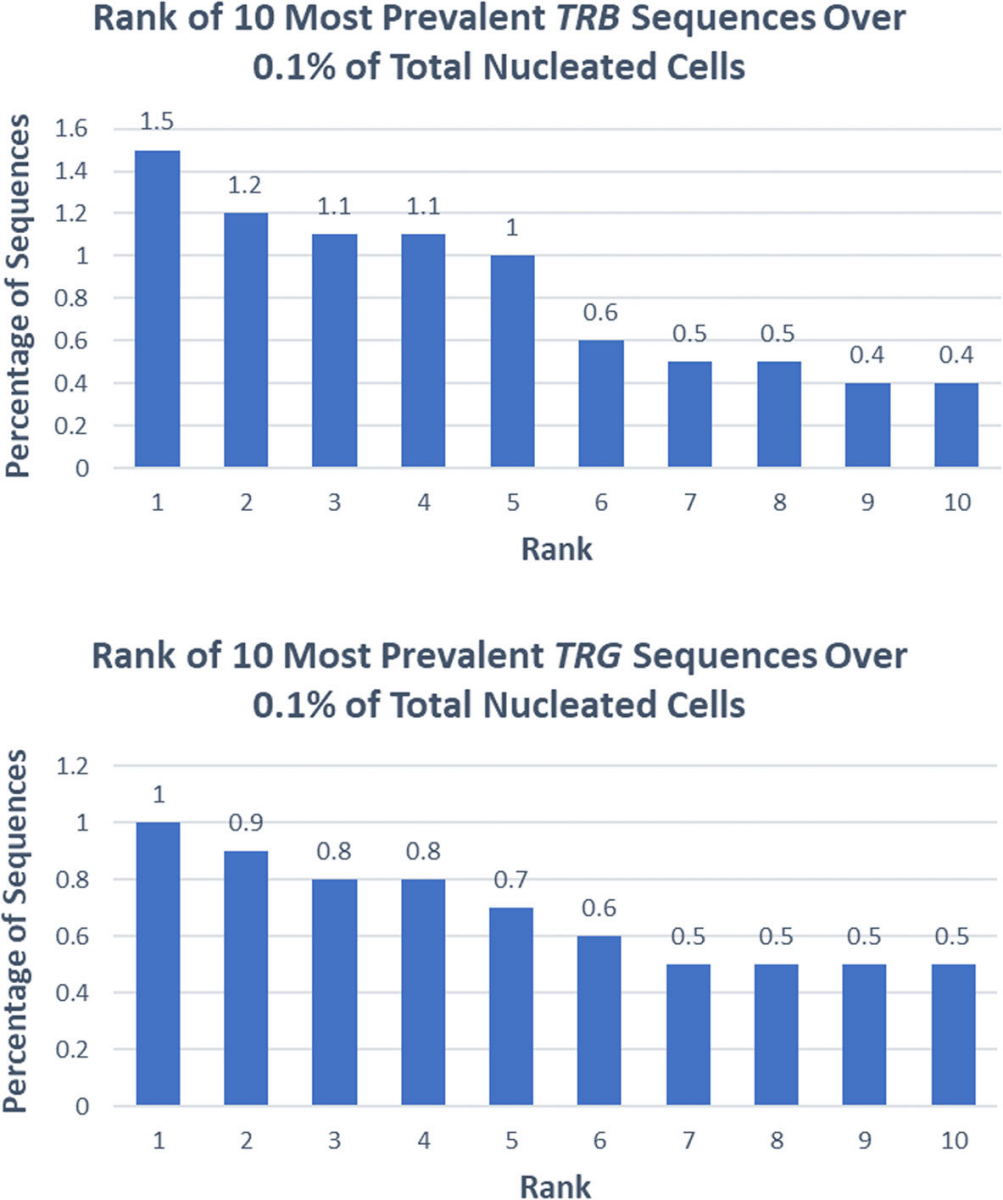
Case 2. Rank of most prevalent sequences over 0.1% by next-generation sequencing of *TRB* (top) and *TRG* (bottom) for one skin biopsy

**Fig. 9:**
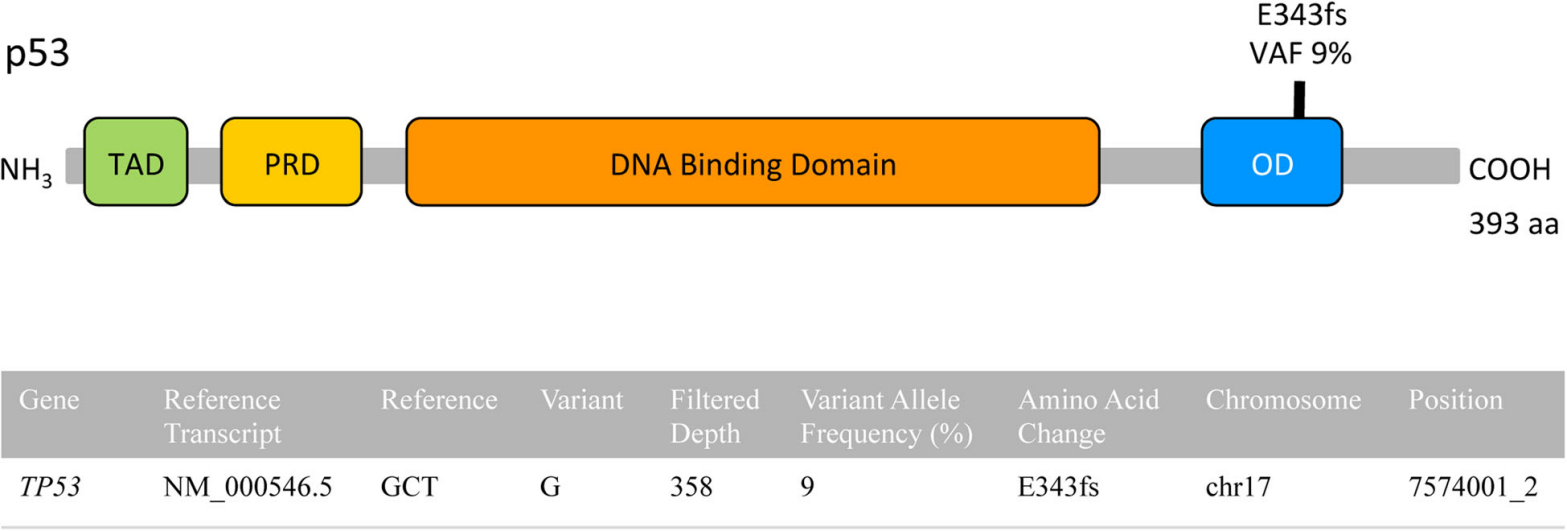
Candidate pathogenic mutation in patient 2. Top: Structure of p53 protein with binding domains and frameshift mutation at position 343 located in the oligomerization domain. TAD = Transactivation domain (green), PRD = proline rich domain (yellow), OD = oligomerization domain (blue). Bottom: Candidate pathogenic mutation identified via targeted sequencing analysis of case 2 biopsy specimen by Heme-STAMP. Filtered depth = read depth of the variant allele

A 69-year-old male was referred to our institution with a 13-year history of progressive hypertrophic and eroded plaques present diffusely over the face, scalp, and entire body. Multiple biopsies showed epidermal hyperplasia with erosion and a dense lichenoid lymphocytic infiltrate that progressed over time to include a more significant epidermotropic component. Upon presentation at our institution, erosions were present on the posterior oropharynx and mucosal lip, and widely distributed ulcerated plaques and tumors were present on the face, scalp, back, and posterior arms (Fig. 6a-b). His treatments included total skin electron beam therapy, bexarotene, brentuximab, pembrolizumab, and pralatrexate with variable clinical response (Fig. 6c-d). His biopsies (a total of 17) showed acanthosis and mild spongiosis with dyskeratotic keratinocytes and an underlying dense lichenoid infiltrate of mostly small to medium-sized lymphocytes with convoluted nuclei extending around adnexal structures into the mid and deep dermis (Fig. 7a). Epidermotropism of haloed atypical lymphocytes including in intraepidermal clusters was increasingly prominent in his later biopsies, which also showed a progressively greater component of large atypical lymphocytes reflective of large cell transformation (Fig. 7b). Immunohistochemistry also showed a predominance of CD3-positive T-cells expressing TCRß, with a CD4:CD8 ratio of at least 10:1 (Fig. 7c-e), with retained CD2 expression and partial loss of T-cell antigens CD5 and CD7. A subset of the cells (up to 15%) expressed CD30 (Fig. 7f). PD-1 was negative. Immunohistochemistry for TIA1 and granzyme was positive in a subset of T-cells, CD56 was negative, and CD20 and MUM1 highlighted background B-cells. No TCR clonality was detected by NGS of 6 biopsies (Fig. 8). A biopsy specimen was sent for targeted NGS, which revealed a pathogenic mutation in *TP53* (c.1028_1029del, p.E343fs*3, NM_000546.5, VAF 9%) (Fig. 9). This mutation is predicted to result in a frameshift and early termination codon leading to loss of function of the tumor suppressor p53 [17]. *TP53* is the most commonly mutated gene in human cancer, and mutations are typically associated with poor prognosis. This clonal mutation provides definitive support for classification as CTCL. The patient ultimately died due to complications of his disease.

## Discussion and conclusions

We present two cases of CTCL with pathogenic somatic mutations in the setting of consistently polyclonal TCR genes. Based on the clinical and histologic findings and the presence of pathogenic somatic mutations, we conclude that these cases are examples of true T-cell lymphomas with repeatedly negative T-cell receptor clonality studies performed by both conventional PCR as well as NGS. There are several potential hypotheses for why the TCR clonality assays are negative in these cases. One possibility is that the TCR genes are in a germline configuration and that these represent natural killer (NK)-cell lymphomas. However, immunohistochemical stains show that in both cases the infiltrates consist of numerous TCRβ-positive, CD4-positive T-cells and that CD56 highlights, at most, a minority of the infiltrate. These findings strongly argue against NK-cell lymphoma. Another possible hypothesis is lack of sensitivity of the clonality assays. This is an unlikely contributor, however, as PCR-based clonality assays demonstrate a sensitivity of 0.1 to 1% [18] and NGS of *TRB* and *TRG* has even greater sensitivity and specificity than PCR [19]. Based on the histologic features and the VAFs of the pathogenic mutations in these cases (between 6 and 9%), it is highly unlikely that a paucity of lymphoma cells or low proportion of lymphoma explains the negative clonality studies. We also considered that the negative TCR clonality in these cases is due to mutations that result in impaired primer binding or deletions, insertions, or rearrangements that otherwise affect the *TRB* and *TRG* loci and prevent proper amplification during the PCR step of the assay. Also, some rare gene rearrangements may not be detected due to lack of included primers for rare V genes or due to disruption of primer binding sites by rare germline variants. We utilized an orthogonal method of RNAseq in one patient to test whether the lack of clonal rearrangement could be due to incompatibility with PCR based assays. Consistent with the clonality assays, we found polyclonal expression of *TRB* and *TRA* variable region genes and no clonal TCR gene rearrangements, although not all rearrangements are expressed. It is possible that these lymphomas may be truly polyclonal with respect to their TCR rearrangements, as has been previously proposed [20, 21]. Although conventional wisdom dictates that *TCD* sequencing would not be useful in this setting since this gene is deleted in αβ T-cells, the potential utility of *TCD* sequencing in similar cases warrants future exploration.

Cases such as the two presented here suggest that a diagnosis of a T-cell malignancy remains possible even with a polyclonal TCR-NGS result and even when the disease burden meets or exceeds the sensitivity threshold of the assay. In these cases, targeted NGS for somatic mutations can help clarify a diagnosis. The benefit of this type of sequencing is manifold. First, identification of pathogenic mutations strongly supports the presence of a neoplastic process. Second, even if no pathogenic mutations can be found, the presence of variants of unknown significance in similar allele frequencies not suggestive of germline variants (i.e. VAF near 50%) may provide a somatic mutation signature that supports the presence of a clonal proliferation. These data can help arrive at a diagnosis of T-cell lymphoma even in the absence of detectable TCR gene rearrangement, and furthermore can help determine prognosis, guide therapy, and potentially monitor disease. For our second patient, mutations in *TP53* have been associated with resistance to several chemotherapeutic medications and are the subject of active research involving small molecule modulators [22]. In the case of our first patient, multiple drugs targeting the PI3K pathway and associated AKT and mTOR pathways are already in clinical use and others are currently in clinical trials [23], making identification of this mutation potentially therapeutically informative. Further, the nodular granulomatous dermal infiltrates seen in this patient’s biopsies share some features with the granulomatous lesions described in activated phosphoinositide 3-kinase delta syndrome, and could suggest possible mosaicism for the *PIK3CD* E1021K mutation.

Given our patients’ pattern of involvement and CD4-positive immunophenotype, diagnostic considerations include mycosis fungoides (MF), Sézary syndrome (SS), T-cell lymphomas with T-follicular helper differentiation, such as angioimmunoblastic T-cell lymphoma or peripheral T-cell lymphoma with T follicular helper differentiation, and peripheral T-cell lymphoma, not otherwise specified. The absence of PD-1 expression argues against a T-cell lymphoma with T-follicular helper differentiation. Also, as at least 90% of Sezary syndromes and many cases of mycosis fungoides express this marker, absence of PD-1 expression and expression of cytotoxic markers raise the possibility that these two cases are phenotypically distinct from typical MF/SS cases [24–26].

To our knowledge, these two highly unusual cases are the first examples of using somatic mutational profiling to detect clonal populations in cutaneous T-cell lymphomas that lack detectable clonal T-cell receptor gene rearrangement. With study of additional cases, mutational profiling of abnormal T-cell lymphoproliferations may prove a useful diagnostic adjunct to results of clinical and histopathologic examination and TCR gene rearrangement testing.

## Data Availability

The datasets used and/or analyzed during the current study are available from the corresponding author on reasonable request.

## Abbreviations

CTCL: Cutaneous T-cell lymphoma
EBV: Epstein-Barr virus
Heme-STAMP: Solid Tumor Actionable Mutation Panel for Hematopoietic and Lymphoid Malignancies
MF: Mycosis fungoides
MRD: Minimal residual disease
NGS: Next-generation sequencing
NK: Natural killer
PCR: Polymerase chain reaction
SS: Sézary syndrome
TCR: T-cell receptor
*TRB*: T-cell receptor beta gene
*TRG*: T-cell receptor gamma gene
VAF: Variant allele frequency
